# The impact of work-related risk factors on the development of neck and upper limb pain among low wage hotel housekeepers in Gondar town, Northwest Ethiopia: institution-based cross-sectional study

**DOI:** 10.1186/s12199-019-0779-7

**Published:** 2019-05-03

**Authors:** Sintayehu Daba Wami, Awrajaw Dessie, Daniel Haile Chercos

**Affiliations:** Department of Environmental and Occupational Health and Safety, Institute of Public Health, University of Gondar, Gondar, Ethiopia

**Keywords:** Neck pain, Upper limb pain, Musculoskeletal disorders, Hotel housekeepers, Gondar, Ethiopia

## Abstract

**Background:**

Musculoskeletal disorders are a major source of disability accounting for considerable economic loss globally. Studies showed that housekeepers suffer from exposure to many high-risk factors for neck and upper limb musculoskeletal disorders. In Ethiopia, little is known and the information is limited in scope about the magnitude of the problem among hotel housekeepers. Therefore, this study aimed to determine the magnitude of the neck and upper limb musculoskeletal disorders and identify the associated risk factors among hotel housekeepers.

**Methods:**

Institution-based cross-sectional study design was employed from March 1 to May 20, 2017. Systematic random sampling was used to select 422 study participants among the Gondar town hotels, Ethiopia. The standardized Nordic questionnaire for the analysis of musculoskeletal symptoms was used to measure the neck and upper limb musculoskeletal disorders. Bivariate and multivariable binary logistic regression analyses were performed using SPSS version 20. The significance level was obtained at 95% CI and *p* value ≤ 0.05.

**Results:**

The overall magnitude of a self-reported neck and upper limb musculoskeletal disorders among hotel housekeepers in the last 12 months was 62.8% (95% CI 58.3, 67.8). The main body areas of concern were neck pain (50.7%), shoulder pain (54%), elbow/forearm (47.2%), and hand/wrist (45.5%). Age, rest break taken, repetitive movement, reaching/overstretching, organization concern for health and safety, and job satisfaction were the risk factors significantly associated with neck and upper limb musculoskeletal disorders.

**Conclusions:**

A higher proportion of hotel housekeepers were found to be affected by neck and upper limb musculoskeletal disorders in Gondar town. Repetitive movement and reaching/overstretching were strongly associated risk factors with neck and upper limb musculoskeletal disorders. Therefore, ergonomic, organizational and personal measures, which focus on minimizing repetitive movement and awkward working position and facilitating rest break with exercise, are important to tackle neck and upper limb musculoskeletal disorders among hotel housekeepers.

## Background

Neck and upper limb musculoskeletal disorders are the most common health problem in the workplace accounting for considerable economic loss [1–7]. Musculoskeletal disorders (MSDs) are impairments of body structures such as tendons, muscles, joints, ligaments, nerves, bones, or a localized blood circulation system caused or aggravated by the work [7–10]. While neck and upper limb musculoskeletal disorders are defined as conditions which affect the soft tissues (tendons, muscles, joints, ligaments, and nerves) of the neck and upper limbs [9, 11–13].

Housekeeping is a physically demanding job that includes many tasks and housekeepers suffer from exposure to many high-risk factors for neck and upper limb MSDs [14]. Many of the tasks are repetitive in nature such as bed making, buffing and vacuuming, emptying garbage, tidying, dusting, and cleaning floors. Moreover, housekeepers are engaged in push-pull task that result biomechanical strain factors such as joint torque, compressive and shear forces, and their influencing variables like specific muscle activity, body positioning, the direction of exertion, and workspace environment [13, 15].

Studies conducted across countries showed a high magnitude of the neck and upper limb MSDs among housekeepers [13, 16–18]. Despite the high magnitude of the problem, little is known and the information is limited in scope about the magnitude and associated factors of neck and upper limb MSDs among hotel housekeepers in Africa [16].

Adverse events due to neck and upper limb MSDs represent a major source of disability globally and have a significant socio-economic impact [2, 19, 20]. Moreover, employees with neck and upper limb MSDs experience physical and mental suffering and temporary or permanent limitations in their professional activities [21]. Many factors can be associated with neck and upper limb MSDs. Different studies conducted showed that repeated lifting of heavy objects, prolonged bending of the neck, overstretching, low little job control, and low supervisor support are significantly associated factors with neck and upper limb MSDs [16, 22, 23]. Furthermore, Neck and upper limb MSDs were not only associated with physical workplace factors but also with psychosocial factors like stress, lack or poor communication, and work ambiguity [24–26].

There is strong evidence that technical ergonomic measures and several kinds of interventions including organizational and personal measures can reduce the occurrence of neck and upper MSDs [7, 27–32]. However, in most African countries including Ethiopia, little is known about the risk factors associated with neck and upper limb MSDs among hotel housekeepers. There is also little empirical evidence about the magnitude of the problem in Ethiopia.

Therefore, this study aimed to determine the magnitude of neck and upper limb MSDs and identify the associated risk factors among hotel housekeepers.

## Methods

### Aim, study design, and setting

The overall aim of this study was to assess the magnitude of neck and upper limb MSDs and associated risk factors among hotel housekeepers in Gondar town, Northwest Ethiopia.

Institution-based cross-sectional study design was employed from March 1 to May 20, 2017. The study was conducted in Gondar town hotels, Northwest Ethiopia. Gondar town is one of a tourist destination city in Amhara Regional State, which is located in the northwestern part of Ethiopia, about 750 km from the capital city, Addis Ababa. A hotel industry was one of the known industries in the town. There are more than 120 hotels in the town, and housekeepers are the largest workforce in this industry.

### Source and study population

All hotel housekeepers in Gondar town were the source population. Those housekeepers who had worked at least for 12 months were included in this study. While hotel housekeepers with spinal deformities or accidents affecting the musculoskeletal system were excluded from the study.

### Sample size determination and sampling procedures

The sample size was determined by using a single population proportion formula, assuming a 50% proportion of neck and upper limb MSDs, 5% margin of error and 95% confidence interval. By considering possible non-response during the data collection period a 10% non-response rate was added on the final sample size. Finally, a total of 422 study participants were included in the study. A systematic random sampling technique was used to select the study participants among the hotels.

### Data collection tool and procedures

The data was collected through face-to-face interview data collection technique.

A standardized Nordic questionnaire for the analysis of musculoskeletal symptoms was used to measure the outcome variable, neck, and upper limb MSDs. The questionnaire was designed to assess musculoskeletal trouble occur in a given population with consideration of in which parts of the body they are localized. The reliability of the questionnaire has been shown to be acceptable [33]. Moreover, socio-demographic, personal, and work environment characteristics of the participants were also collected.

The questionnaire was originally in the English version, and it was translated to Amharic local language and back to English by another translator to check the consistency of message from the question. The translation was then reviewed by professional experts. Prior to the commencement of actual data collection, the questionnaire was pretested in 42 (10%) of study subject in Woreta town hotel housekeepers and necessary modification was made on the tool. Six Bachelor of Science graduate of Environmental and Occupational health and safety’ data collectors with three supervisors were assigned for the data collection.

### Operational definitions

#### Neck trouble

Musculoskeletal symptoms in the neck were defined by aches, pain, or discomfort during the 12 months preceding completion of the questionnaire.

#### Upper limb/extremity musculoskeletal disorders

Musculoskeletal symptoms experienced in the upper limb (shoulders, elbows, hands, or wrists) irrespective of neck pain were defined by aches, pain, or discomfort in the past 12 months were also used as an outcome measure.

#### Body mass index

Weight in kilograms divided by the square of the height in meters (kg/m^2^).

- Underweight = BMI < 18.50
- Normal range = BMI b/n 18.50–24.99
- Overweight = BMI b/n 25.00–29.99
- Obese = BMI ≥ 30.00

#### Satisfaction

The employee was considered satisfied with a job when his/her sum of generic job satisfaction scale score was 32 or above [34].

### Data management and analysis

Data was checked, edited, coded, and entered to Epi-info version 7.00 and exported to Statistical Package for Social Science (SPSS) version 20 for further analysis.

A chi-square test was conducted to see the association of different factors with the magnitude of neck and upper limb MSDs. A binary logistic regression model was fitted to identify factors associated with neck and upper limb MSDs. Neck and upper limb MSDs were regressed against the socio-demographic, personal, and work environment factors separately. Before fitting the binary logistic regression model, first the goodness of model fit test was checked by the Hosmer-Lemeshow test, and the assumption was satisfied (*p* value > 0.05).

Bivariate binary logistic regression analysis was performed and variable with *p* value < 0.20 was exported to multivariable binary logistic regression analysis. The significance level was obtained at 95% CI and *p* value ≤ 0.05. The adjusted odds ratio was used to determine the strength of association.

### Data quality control

During the collection of data, each completed questionnaire was checked for consistency and completeness by supervisors. Throughout the course of the data collection, data collectors were supervised at each site, regular meetings were held between the data collectors and the principal investigator. Ten percent of data was double entered to check error during data entry.

## Results

All 422 completed and valid questionnaires were returned and considered for the analysis, which gives a response rate of 100%.

### Socio-demographic and personal characteristics of the study participants

From the total respondents, majority 388 (91.9%) were females. The mean (±SD) age of the respondents was 26.71 ± 4.9 years and the age of the study participants ranged from 20 to 40 years. Almost all 397 (94.1%) of respondents do not perform physical activity/exercises and 64 (15.2%) of participants were overweight (Table 1).

**Table 1:** Socio-demographic and personal characteristics of the study participants, Northwest Ethiopia, 2017 (*n* = 422)

Variables	Frequency (*n*)	Percent (%)
Sex		
Female	388	91.9
Male	34	8.1
Age (years)		
≤ 24	194	46
25–29	100	23.7
> 29	128	30.3
Marital status		
Single	206	48.8
Married	179	42.4
Divorced/widowed	37	8.8
Educational status		
Illiterate	155	36.7
Primary school	240	56.9
Secondary school and above	27	6.4
Employment pattern		
Permanent	378	89.6
Temporary	44	10.4
Specific work experience in this work area		
1–2 years	291	69
> 2 years	131	31
Monthly salary (ETB)		
≤ 500	96	22.7
501–1000	267	63.3
> 1000	59	14
Body mass index (BMI)		
Underweight	71	16.8
Normal weight	277	65.6
Over weight and	64	15.2
Obese	10	2.4
None work physical activity		
No	397	94.1
Yes	25	5.9
Smoking behavior		
None	417	98.8
Past smoker	3	0.7
Current	2	0.5
Alcohol drinking		
No	314	74.4
Yes	108	25.6

*ETB* Ethiopian birr

### Working condition of respondents

Among the study participants, 71 (16.8%) of respondents were working for more than 8 h per day and only 90 (21.3%) of participants took more than 45 min rest break per day excluding lunch break. More than half, 220 (52.1%) of the respondent's job involve repetitive movements and 248 (58.8%) of the study participants were not satisfied with their current job. More than two third, 307 (72.7%) of respondents’ mentioned that their organizations do not have a concern for the health and safety of the workers (Table 2).

**Table 2:** Working conditions of the study participants, Northwest Ethiopia, 2017 (*n* = 422)

Variables	Frequency (*n*)	Percent (%)
Hours worked per day		
≤ 8 h	351	83.2
> 8 h	71	16.8
Rest break taken per day (excluding lunch break)		
< 30 min	107	25.4
30–45 min	225	53.3
> 45 min	90	21.3
The job requires repetitive work		
No	202	47.9
Yes	220	52.1
The job requires reaching/overstretching		
No	201	47.6
Yes	221	52.4
The job requires bending		
No	204	48.3
Yes	218	51.7
Satisfaction		
No	248	58.8
Yes	174	41.2
Training		
No	323	76.5
Yes	99	23.5
Manager concern for health and safety		
Disagree/strongly disagree	307	72.7
Neutral	93	22
Agree/strongly agree	22	5.2

### The magnitude of neck and upper limb musculoskeletal disorders

The finding of this study revealed that the overall magnitude of self-reported neck and upper limb MSDs among hotel housekeepers was 62.8% (95% CI 58.3, 67.8). The main body areas of concern were neck pain (50.7%), shoulder pain (54%), elbow/forearm (47.2%), and hand/wrist (45.5%). Moreover, 58.2% of respondents were prevented from doing their normal work because of the neck and upper limb disorders from 1 to more than 30 days. Furthermore, the length of time they had neck and upper limb pain also ranges from 1 to more than 30 days.

### Factors associated with neck and upper limb pain

The multivariable binary logistic regression showed that rest break taken, repetitive movement, reaching/overstretching, and satisfaction had statistically significant association with neck pain at *p* value < 0.05 (Table 3). While age, repetitive movement, overstretching/reaching, satisfaction, and organization concern for health and safety had statistically significant association with upper limb pain at *p* value < 0.05 (Table 4).

**Table 3:** Bivariate and multivariable binary logistic regression analysis of factors associated with neck trouble among hotel housekeepers, Northwest Ethiopia, 2017 (*n* = 422)

Variables	Neck pain		COR (95% CI)	AOR (95% CI)
	No	Yes		
Sex				
Male	26	8	1.00	1.00
Female	182	206	3.68 (1.62, 8.33)^*^	2.1 (0.82, 5.42)
Age (years)				
≤ 24	102	92	1.00	1.00
25–29	45	55	1.36 (0.84, 2.2)	1.32 (0.72, 2.43)
> 29	61	67	1.22 (0.78, 1.9)	0.9 (0.5, 1.59)
Educational status				
Illiterate	84	71	1.00	1.00
Primary school	109	131	1.42 (0.95, 2.13)	1.19 (0.73, 1.96)
Secondary school and above	15	12	0.95 (0.42, 2.15)	0.76 (0.27, 2.12)
Specific Work experience in this work area				
1–2 years	151	140	1.00	1.00
> 2 years	57	74	1.4 (0.93, 2.12)	1.06 (0.64, 1.77)
Body mass index (BMI)				
Under weight	31	40	1.00	1.00
Normal weight	145	132	0.71 (0.42, 1.19)	1.14 (0.0.59,2.19)
Over weight and obese	32	42	1.02 (0.53, 1.96)	1.62 (0.71, 3.67)
Rest break taken per day (excluding lunch break)				
< 30 min	37	70	1.00	1.00
30–45 min	104	121	0.62 (0.38, 0.99)*	0.71 (0.39, 1.26)
> 45 min	67	23	0.18 (0.1, 0.337)**	0.29 (0.13, 0.63)^*^
The job requires repetitive work				
No	146	56	1.00	1.00
Yes	62	158	6.6 (4.34, 10.17)**	1.98 (1.01, 3.87)^*^
The job requires reaching/overstretching				
No	149	52	1.00	1.00
Yes	59	162	7.87 (5.1, 12.15)**	3.72 (1.81, 7.66)^**^
The job requires bending				
No	135	69	1.00	1.00
Yes	73	145	3.89 (2.59, 5.82)**	0.64 (0.31, 1.29)
Satisfaction				
No	82	166	1.00	1.00
Yes	126	48	0.19 (0.12, 0.29)**	0.46 (0.27, 0.77)^*^
Training				
No	140	183	1.00	1.00
Yes	68	31	0.35 (0.22, 0.56)**	0.57 (0.31, 1.04)
Manager concern for health and safety				
Disagree/strongly disagree	154	153	1.00	1.00
Neutral	36	57	1.59 (0.99, 2.56)	0.82 (0.45, 1.50)
Agree/strongly agree	18	4	0.22 (0.07, 0.68)*	0.31 (0.08, 1.17)

Gender, sex, and BMI were adjusted covariates

Note: *1:00* = reference, * = variable *p* value < 0.05, ** = *p* value ≤ 0.001

**Table 4:** Bivariate and multivariable binary logistic regression analysis of factors associated with upper limbs pain among hotel housekeepers, Northwest Ethiopia, 2017 (*n* = 422)

Variables	Upper limb pain		COR (95% CI)	AOR (95% CI)
	No	Yes		
Sex				
Male	25	9	1.00	1.00
Female	146	242	4.6 (2.09, 10.14)**	2.64 (0.98, 7.09)
Age (years)				
≤ 24	96	98	1.00	1.00
25–29	30	70	2.29 (1.37, 3.81)*	3.39 (1.63, 7.07)*
> 29	45	83	1.8 (1.14, 2.86)*	1.56 (0.81, 2.99)
Educational status				
Illiterate	72	83	1.00	1.00
Primary school	88	152	1.49 (0.99, 2.26)	1.15 (0.66, 2.02)
Secondary school and above	11	16	1.26 (0.55, 2.89)	0.97 (0.31, 3.11)
Specific work experience in this work area				
1–2 years	122	169	1.00	1.00
> 2 years	49	82	1.2 (0.79, 1.84)	0.78 (0.43, 1.41)
Body mass index (BMI)				
Under weight	26	45	1.00	1.00
Normal weight	118	159	0.78 (0.45, 1.33)	1.89 (0.0.88,4.08)
Over weight and obese	27	47	1.01 (0.51, 1.98)	1.99 (0.76, 5.20)
Rest break taken per day (excluding lunch break)				
< 30 min	31	76	1.00	1.00
30–45 min	89	136	0.62 (0.38, 1.02)	0.61 (0.31, 1.23)
> 45 min	51	39	0.31 (0.17, 0.56)**	0.47 (0.2, 1.14)
The job requires repetitive work				
No	138	64	1.00	1.00
Yes	33	187	12.22 (7.6, 19.6)**	6.44 (2.99, 13.84)**
The job requires reaching/overstretching				
No	134	67	1.00	1.00
Yes	37	184	9.9 (6.28, 15.74)**	3.33 (1.48, 7.51)*
The job requires bending				
No	117	87	1.00	1.00
Yes	54	164	4.08 (2.7, 6.18)**	0.45 (0.19, 1.05)
Satisfaction				
No	50	198	1.00	1.00
Yes	121	53	0.11 (0.07, 0.17)**	0.22 (0.13, 0.38)**
Training				
No	119	204	1.00	1.00
Yes	52	47	0.53 (0.34, 0.83)*	0.78 (0.41, 1.50)
Manager concern for health and safety				
Disagree/strongly disagree	117	190	1.00	1.00
Neutral	36	57	0.98 (0.61, 1.57)	0.35 (0.17, 0.72)*
Agree/strongly agree	18	4	0.14 (0.05, 0.41)**	0.11 (0.02, 0.60)*

Gender, sex, and BMI were adjusted covariates

Note: *1:00* = reference, * = variable *p* value < 0.05, ** = *p* value ≤ 0.001

#### Neck pain

Rest break taken was positively associated with neck pain. Respondents who had taken more than 45 min rest period per day had 71% less likely odds of having neck pain when compared to participants who had taken less than 30 min rest period per day (AOR = 0.29, 95% CI 0.13, 0.63). Moreover, respondents whose job require repetitive movement had almost two times higher odds of developing neck trouble than whose task does not require repetitive movement (AOR = 1.98, 95% CI 1.01, 3.87). This study showed jobs which require reaching/overstretching were a risk factor for neck trouble. Respondents whose job require reaching/overstretching had 3.72 times higher odds of having neck trouble than those whose task does not require reaching/overstretching (AOR = 3.72, 95% CI 1.81, 7.66). Moreover, respondents who were satisfied with their current job had 54% less likely odds of having neck trouble when compared to those who were not satisfied (AOR = 0.46, 95% CI 0.27, 0.77).

#### Upper limb pain

Age was significantly associated with upper limb disorders. Participants with the age group of 25–29 had 3.39 times higher odds of developing upper limb pain when compared to participants with the age ≤ 24 (AOR = 3.39, 95% CI 1.63, 7.07). Moreover, respondents whose job requires repetitive movement had 6.44 times higher odds of having upper limb pain than whose task does not require repetitive movement (AOR = 6.44, 95% CI 2.99, 13.84). A job that involves overstretching was significantly associated with upper limb pain. Respondents whose job require reaching/overstretching had 3.33 times higher odds of having upper limb pain when compared to those whose task does not require reaching/overstretching (AOR = 3.33, 95% CI 1.48, 7.51). Moreover, respondents who were satisfied with their current job had 78% less likely odds of developing upper limb pain than those who were not satisfied (AOR = 0.22, 95% CI 0.13, 0.38). Furthermore, manager concern for health and safety had a positive association with upper limb pain. Respondents who strongly agree/agree that there was manager concern for health and safety had 89% less likely odds of having upper limb pain than those who strongly disagree/disagree (AOR = 0.11, 95% CI 0.02, 0.60).

## Discussion

The result of this study revealed that the overall magnitude of a self-reported neck and upper limb trouble among Gondar town hotel housekeepers was 62.8% (95% CI 58.3, 67.8). The highest magnitude of MSDs was reported in shoulder body region (54%); followed by neck pain (50.7%), elbow/forearm pain (47.2%), and hand/wrist pain (45.5%) respectively. This result showed that the magnitude of neck and upper limb MSDs among hotel housekeepers in Gondar town was high. This may be explained as hotel housekeepers in this study perform cleaning, washing, and bed dressing which involves lifting, pulling and carrying a heavy load, working with the arms above shoulder, repetitive movement, and working in a range of awkward posture they are at high risk of developing neck and upper limb MSDs [14]. This result is supported by a study conducted in India which stated that the high magnitude of neck and upper limb MSDs among hotel housekeepers may be related to repetitive work, manual handling, and altered posture [27]. The overall magnitude of neck and upper limb MSDs of this study is lower when compared with the study reported, 77% musculoskeletal pain among low-income community female homemakers in Lebanon [35]. The possible reason might be due to the difference in the study participants’ socio-demographic characteristics, and this study only reported neck and upper limb MSDs while they had reported any musculoskeletal pain in the past 12 months.

However, the magnitude of neck and upper limb MSDs of this study is higher when compared with the study reported 32% upper extremity MSDs among hotel housekeepers in the USA (16), and the study that showed neck pain (29.9%), elbow pain (25.4%), and hand/wrist (22%) among hotel housekeepers in Orlando [17]. Another similar research reported 33% neck pain, 23% shoulder pain and 22% hand/wrist pain among cleaner in the UK [36]. However, 74% of cleaners in the later study reported experiencing aches, pains, and discomfort in the last 1 year including back and lower extremity troubles [36]. This difference might be due to the difference in work organization between countries. Those countries might have better work condition, ergonomic design of tools and equipment and organizational concern for health and safety of housekeepers.

Moreover, the magnitude of neck and upper limb MSDs of this study is higher when compared to the study reported, 33.3% neck pain, 23.3% shoulder pain, 36.7% hand/wrist pain and 8.3% elbow pain among full time lady servants in Egypt [16], and 42% shoulder pain, 35% neck pain, 29% hand/wrist pain, and 18% elbow pain among housewives in India [37]. This difference might be due to the difference in the sample size of the studies, socio-demographic characteristics, and organization of the work. Other possible reasons might be due to the prolonged work without the opportunity to rest and tasks carried by hotel housekeepers. In this study, housekeepers’ job involves more awkward posture, repetitive movement of the neck and upper extremity, and perform less physical exercise. In addition, there is less concern of organization for health and safety of housekeepers compared with those countries.

The multivariable binary logistic regression showed that there was no significant association observed between sex, body mass index (BMI), physical exercise, and neck and upper limb MSDs. However, different studies reported the presence of gender difference in upper extremity MSDs among the working population in many occupational classes, with female workers having a higher risk [38–40]. This difference might be due to the differences in the distribution of male and female workers in occupations with different risks for the neck and upper limb MSDs between countries.

Rest break taken had statistically significant association with neck pain. Respondents who had taken more than 45min rest period per day had 71% less likely odds of having neck pain when compared to participants who had taken less than 30 min rest period per day. This result may be explained as; when workers take more frequent rest breaks in working day possibility of recovery increased and reduce the incidence of neck pain. This is in line with the European agency of health and safety report which stated that prolonged work without the opportunity to rest and recover from the load is a risk factor for MSDs [41].

This study showed that works which involve a repetitive movement of the neck and upper limb had a statistically significant association with neck and upper limb trouble. Participants whose job require repetitive movement of upper extremities’ had higher odds of having neck and upper limb MSDs than those whose task does not require repetitive movement. This finding is in line with the result reported in Egypt [16], the UK [22], Lebanon [35], and the USA [42]. This might be explained as work involving repetitive movements is very tiring because the worker cannot fully recover in the short periods of time between movements, thus neck and upper limb MSDs might develop. Moreover, as repetitive movement involves the same joints and muscle groups, the risk of neck and upper limb MSDs increased.

This study found that jobs which require reaching/overstretching were a risk factor for neck and upper limb MSDs. Respondents whose job require reaching/overstretching had almost 3.5 times higher odds of having neck and upper limb MSDs than those whose task does not require reaching/overstretching. This result is in line with the study conducted in Egypt [16], France [24], and Lebanon [35], which reported working in awkward posture including working with hands above shoulder was a risk factor for neck and upper limb MSDs. The possible reason might be hotel housekeepers usually working in uncomfortable working posture or awkward postures represent unnatural positions, deviated from “neutral positions,” in which joints are held or moved away from the body’s natural position. The closer the joint is to its end of the range of motion, the greater the stress placed on the soft tissues of that joint, such as muscles, nerves, and tendons. When muscles are contracted, the body is subjected to a greater mechanical effort. When working with joint positions of the upper limb outside comfort angle; increase the possibility of upper limb MSDs.

According to this study, hotel housekeepers who were satisfied with their current job were less likely to be affected by neck and upper limb MSDs when compared to those who were not satisfied. This finding is similar to the study conducted in the UK [22] which reported poor job satisfaction was associated with the increased magnitude of neck and upper limb MSDs. This finding is also supported by a study conducted in Quebec [43], which reported cleaners working alone may feel isolated even isolated onwards with others, so this might lead to less satisfaction and increase the risk of MSDs. Other possible explanations might be when they work in a situation with high job satisfaction, a high influence over work-related decisions and get social support they are less likely to develop neck and upper limb MSDs than others [44].

As per this study, age was significantly associated with upper limb disorders. Participants with the age group of 25–29 had 3.39 times higher odds of having upper limb pain when compared to participants with the age ≤ 24. This finding is in line with the study conducted in Seoul hotel workers [39] and France [24] which reported the risk of developing upper limb MSDs was higher among older age workers than those with younger age. This might be explained as the functional capacity of soft tissues and muscle strength decrease with age the possibility of upper limb MSDs increase. Other possible reasons might be the accumulation of years of exposure to MSDs risk factors among older age increase the magnitude of upper limb MSDs [23].

Furthermore, manager concern for health and safety had a positive association with upper limb MSDs. Respondents who strongly agree/agree that there was manager concern for health and safety had 89% less likely odds of having upper limb pain than those who strongly disagree/disagree. This might explain how strong support and concern from the manager toward health and safety of hotel housekeepers reduce the risk of upper limb MSDs.

## Limitations of the study

Though this study was able to provide important data on the neck and upper limb pain among hotel housekeepers, several limitations are noted. Not undertaking ergonomic audits of workstations and activities was one of the limitations of the study. In addition, since this study was a questionnaire-based cross-sectional study, the possibility of recall bias could not be ruled out since more serious and recent pains or troubles remembered better than less serious and older one. But we have tried to minimize the effect by honestly explaining the objective and significances of the study to the study participants and by using standardized questionnaire for assessing MSDs. Despite these limitations, we feel the study provide a reasonably accurate assessment of the impact of work-related risk factors on the development of neck and upper limb pain among hotel housekeepers.

## Conclusions

A higher proportion of hotel housekeepers were found to be affected by neck and upper limb musculoskeletal disorders in Gondar town.

Age, rest period taken, repetitive movement, reaching/overstretching, organization concern for health and safety, and satisfaction were the work-related risk factors significantly associated with neck and upper limb MSDs.

Therefore, ergonomic, organizational, and personal measures, which focus on minimizing repetitive movement and awkward working position and facilitating rest break with exercise, are important to reduce neck and upper limb musculoskeletal disorders among hotel housekeepers.

## Abbreviations

AOR: Adjusted odds ratio
BMI: Body mass index
CI: Confidence interval
COR: Crude odds ratio
ETB: Ethiopian birr
MSDs: Musculoskeletal disorders
SPSS: Statistical Package for Social Science
UK: United Kingdom
